# Integrative Analysis of Transcriptomic and Proteomic Changes Related to Cytoplasmic Male Sterility in Spring Stem Mustard (*Brassica juncea* var. tumida Tsen et Lee)

**DOI:** 10.3390/ijms23116248

**Published:** 2022-06-02

**Authors:** Ying Shen, Jie Wang, Rui Xia, Minyang Tong, Yunping Huang, Liai Xu, Zhujun Zhu, Qiufeng Meng, Youjian Yu

**Affiliations:** 1 Collaborative Innovation Center for Efficient and Green Production of Agriculture in Mountainous Areas of Zhejiang Province, College of Horticulture Science, Zhejiang A&F University, Hangzhou 311300, China; 2020101032015@stu.zafu.edu.cn (Y.S.); 2020101032021@stu.zafu.edu.cn (R.X.); 2020601041038@stu.zafu.edu.cn (M.T.); 20210035@zafu.edu.cn (L.X.); zhuzj@zafu.edu.cn (Z.Z.); 2 Ningbo Academy of Agricultural Sciences, Ningbo 315000, China; jieer881018@163.com (J.W.); hyp2003@163.com (Y.H.)

**Keywords:** mustard, transcriptomic analysis, proteomic analysis, cytoplasmic male sterility, carbohydrate, energy metabolism

## Abstract

The development of flower and pollen is a complex biological process that involves multiple metabolic pathways in plants. In revealing novel insights into flower and pollen development underlying male sterility (MS), we conducted an integrated profiling of gene and protein activities in developing buds in cytoplasmic male sterile (CMS) mutants of mustard (*Brassica juncea)*. Using RNA-Seq and label-free quantitative proteomics, 11,832 transcripts and 1780 protein species were identified with significant differential abundance between the male sterile line 09-05A and its maintainer line 09-05B at the tetrad stage and bi-nucleate stage of *B. juncea*. A large number of differentially expressed genes (DEGs) and differentially abundant proteins (DAPs) involved in carbohydrate and energy metabolism, including starch and sucrose metabolism, tricarboxylic acid (TCA) cycle, glycolysis, and oxidoreductase activity pathways, were significantly downregulated in 09-05A buds. The low expression of these DEGs or functional loss of DAPs, which can lead to an insufficient supply of critical substrates and ATP, could be associated with flower development, pollen development, and changes in fertility in *B. juncea*. Therefore, this study provided transcriptomic and proteomic information of pollen abortion for *B. juncea* and a basis for further research on the molecular regulatory mechanism of MS in plants.

## 1. Introduction

Male sterility (MS) refers to the inability to produce functional anthers or pollens, which plays an important role in developing high yielding and vigorous cultivars as an economical and effective system for pollination control [1]. Based on the inheritance, MS is classified into cytoplasmic male sterility (CMS) and genetic male sterility (GMS) [2]. CMS, which has been reported in more than 150 plant species, is a maternally inherited defect of higher plants in the production of functional pollen and it has wide application for hybrid production in crops [3,4]. In general, CMS can occur at different stages during reproductive development and CMS lines have stable sterility, adequate flowering, and crossing abilities [5]. To date, many CMS systems, including *oxa* CMS, *ogu* CMS, *nap* CMS, *tour* CMS, *pol* CMS, *hau* CMS, and *orf220*-type CMS [6,7,8,9], have been found and widely used in different *Brassica* crops, such as mustard (*Brassica juncea*), cabbage (*Brassica oleracea*), and oilseed rape (*Brassica napus*).

In plants, CMS is determined by the mitochondrial genome and associated with a pollen sterility phenotype that can be suppressed or counteracted by nuclear genes known as fertility-restorer genes [10]. Recently, great progress has been made in CMS theoretical research and application of different CMS systems in *Brassica* crops. *MutS HOMOLOG1*, a nuclear gene can control illegitimate recombination in plant mitochondria, whose silencing can mediate mitochondrial *orf220* substoichiometric shifting and cause MS in *B. juncea* [11]. In addition, *BjuA017917*, a non-PPR fertility restoration gene encoding a guanosine nucleotide diphosphate dissociation inhibitor, was proposed to be the candidate gene for fertility restoration of the *oxa* CMS line in *B. juncea* [12]. Studies have also shown that the retrograde signal from the mitochondrial genes such as *orf288* could arrest the differentiation of archesporial cells and caused MS in the *hau* CMS line of *B. napus* [13]. In the SaNa-1A CMS line of *B. napus*, active oxygen is greatly accumulated disturbing ROS balance, and the increase in peroxidase activity in the CMS line might inhibit the biosynthesis of auxin and affect anther development [14]. Furthermore, the protein encoded by the *orf138* gene would accumulate on the mitochondrial membrane, which may interfere with the expression of some key genes, such as *atp6*, *atp8*, and *cox I*, in the electron-transport chain, and inhibit the normal development of anthers in *B. oleracea* [15]. However, key genes and proteins involved in metabolic pathways regarding CMS must be further investigated.

In recent years, transcriptomic sequencing and proteomic integrated analysis have been widely used in plants on pollen development, fertility transition, and biological and abiotic stress responses to gain high-resolution data with great accuracy and efficiency [16,17,18]. They have also been applied to obtain key information regarding pollen development in various CMS mutants of cabbage, cotton, rice, and other crops [19,20,21]. Xing et al. [22] found that the key differentially expressed genes (DEGs) participating in gibberellin-mediated tapetum-programmed cell death (PCD) pathways and sporopollenin biosynthesis were significantly related to the sterility phenotype in *Ogu* CMS line R2P2CMS using integrated RNA-seq and isobaric tags proteomic analysis. By transcriptomic and proteomic analyses, Tang et al. [23] deduced that the low expression of DEGs and differentially abundant proteins (DAPs) involved in energy metabolism, such as *psbS*, *psaD*, and *ATPF1G*, may result in pollen abortion in the male sterile mutant MS2-2 of *Tagetes erecta*. Hao et al. [24] identified several critical regulatory genes including *orf279* which may lead to delayed tapetum PCD, causing pollen abortion in AL18A of a wheat CMS line through combined transcriptomic and proteomic analysis. Using transcriptomic and proteomic analysis, Ning et al. [25] showed that transcription factors related to early anther differentiation, such as *SPOROCYTELESS*, *MYB80*, and *ABORTED MICROSPORES*, at the young bud stage, were downregulated, which affected early anther development in Shaan2A CMS lines. Therefore, combined transcriptomic and proteomic analyses can be a good research method to facilitate the understanding of the molecular regulatory mechanism underlying MS and nuclear mitochondrial interaction in plants.

Mustard (2n = 36, AABB) is an allopolyploid derived from interspecific hybridization between *B. rapa* (2n = 20, AA) and *Brassica nigra* (2n = 16, BB), which is widely grown as a food crop because of its adaptation to varying growth conditions [26,27]. In addition, it is used for medicinal and industrial purposes because of its rich nutrients and bioactive components [28]. *Oxa* CMS was first observed in *B. juncea*, and it is now widely used in increasing the genetic diversity of CMS and promoting the use of heterosis in *Brassica* crops. In the *oxa* CMS of stem mustard, anthers are surrounded by unfolded petals, resulting in early stigma exposure, male sterile pollen grains, and failed self-pollination [8]. The abortive stage of anther development in *oxa* CMS is initiated at the late uninucleate stage and abnormally vacuolated microspores can cause MS [8]. A spring stem mustard CMS line system, named as 09-05A/B, was established using the *oxa* CMS winter mustard and spring mustard maintainer line, and the male sterile line 09-05A shows a stable sterility characteristic with shriveled anthers, and entirely abortive and abnormally degraded pollen grain produced in floral developments [8]. Thus, this CMS system serves as an ideal material to study the molecular regulatory mechanism of pollen development and metabolic pathways regarding CMS in *Brassica* plants.

In order to identify a set of key candidate genes, biological processes, and pathways underlying the MS in *B. juncea*, a systemic understanding of the molecular regulator mechanism and metabolic networks during the pollen development is urgently needed. In the present study, transcriptomic sequencing and label-free proteome were performed using flower buds during the tetrad (TE) stage and bi-nucleate (BI) stage between the male sterile line 09-05A and the maintainer line 09-05B of *B. juncea*, to identify critical DEGs and DAPs related to pollen development. Our research found several key biological processes related to MS, such as “carbohydrate metabolic process”, “oxidoreductase activity”, “sucrose metabolic process”, and “TCA cycle”. The results lay the foundation for exploring the genetic and molecular mechanisms of *B. juncea* pollen development, and provide further knowledge for the development of germplasm innovation and heterosis utilization in plants.

## 2. Results

### 2.1. Phenotypic and Cytological Characterization in 09-05A/B Lines of B. juncea

**Figure 1:**
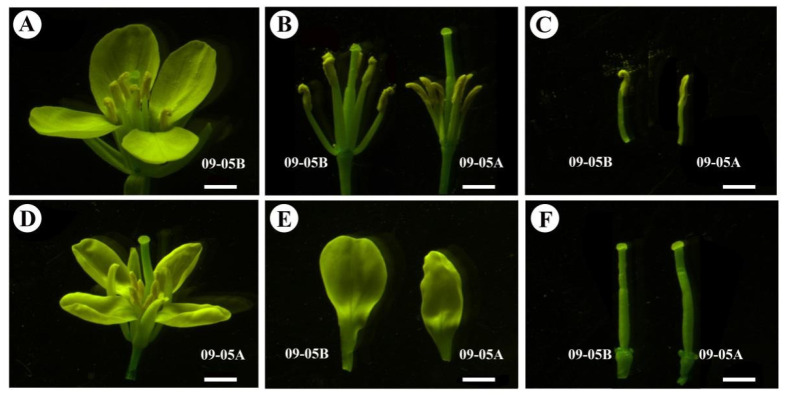
Morphological observation of floral organ between the male sterile line 09-05A and its maintainer line 09-05B of *B. juncea* at the blossom stage. (**A**) The flower bud of 09-05B. (**B**) Comparison of stamens and pistils of 09-05A/B lines. (**C**) Comparison of stamens of 09-05A/B lines. (**D**) The flower bud of 09-05A. (**E**) Comparison of petals of 09-05A/B lines. (**F**) Comparison of pistils of 09-05A/B lines. Bars = 2 mm.

**Figure 2:**
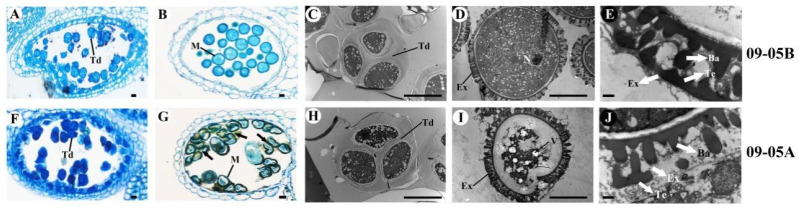
The cross-section analysis observation of anthers and microspores at the TE and BI stages of *B.* *juncea* from 09-05B and 09-05A. (**A**,**F**) Semi-thin section of anthers at the TE stage; (**B**,**G**) semi-thin section of anthers at the BI stage; (**C**,**H**) TEM micrographs of microspores at the TE stage; (**D**,**I**) TEM micrographs of microspores at the BI stage; (**E**,**J**) TEM micrographs of pollen exine at the BI stage. Ba—baculum; Ex—exine; M—microspore; N—nucleus; Td—tetrad; Te—tectum; V—vacuole. Arrows in (**G**) showed the remnants of the aborted microspores. (**A**–**D**,**F**–**I**) Bars = 10 μm; (**E**,**J**) Bars = 0.5 μm.

The morphological and cytological features of 09-05A and 09-05B were compared during flowering. Morphologically, light microscopy showed that 09-05A had a similar flower pattern to its maintainer line 09-05B, and corolla at the blossom stage from 09-05A grew smaller than those from 09-05B (Figure 1A,D). Compared with the yellow and plump anthers of 09-05B, the anthers of 09-05A were longer and shrunken, with no evident pollen grains on the surface (Figure 1B,C). Other floral tissues of 09-05A, such as sepals, petals, and pistil, remained normal (Figure 1D–F). The observation of semi-thin sectioning under the microscope showed that tetrads were formed normally between 09-05A and 09-05B at the TE stage (Figure 2A,F). However, at the BI stage, visible abnormalities appeared in the anther of 09-05A, in which most microspores were shrunken and deformed (Figure 2G) compared with those from 09-05B (Figure 2B). The transverse section observation of anthers showed that, with the morphological abnormalities of microspore cell in 09-05A, a significant microscopic phenotype developed, indicating that the BI stage was the vital period of microspore abortion in 09-05A.

Transmission electron microscopy (TEM) was applied to obtain high-resolution images, comparatively analyze the pollen grains from 09-05A and 09-05B at the TE and BI stages, and comprehensively understand the cellular defects of pollen grains in 09-05A anthers. Consistent with the abovementioned results, the structure of tetrads in 09-05A (Figure 2H) was normal and similar to that in 09-05B (Figure 2C) at the TE stage. The cell nucleus in pollen grains was evident in 09-05B (Figure 2D); however, the cell nucleus and other cellular components were degraded in 09-05A at the BI stage (Figure 2I). Moreover, the bacula and tectum on the pollen exine wall in the microspore of 09-05B were normal (Figure 2E), whereas their morphology was significantly altered in the 09-05A CMS line (Figure 2J).

Based on these results, we concluded that the abortive stage of anther development in 09-05A was initiated after the TE stage, and most pollen grains showed abortion at the BI stage during the mitotic phase. Shrunken and deformed microspores and the lack of viable pollen grains could cause MS during anther development in 09-05A. Therefore, flower buds from the TE and BI stages were used as the transcriptomic and proteomic sequencing material.

### 2.2. Transcriptomic Analysis and Assembly

We selected the TE and BI stages for transcriptomic analysis to explore the molecular regulatory mechanism underlying microspore abortion in 09-05A. Raw reads were filtered to remove low-quality reads, and a total of 552,293,954 clean reads were ultimately used. The percentage of sequences with nucleotide mass fraction Q30 values greater than 30 was 93.73% in all samples, and the GC content was 46.72% (Table S1). By comparing the reads with the reference genome, the genome alignment of each sample was obtained, and the comparison rate was 81.05–95.52%. An overview of the statistics on transcriptomic sequencing and assembly of two stages of pollen development between 09-05A and 09-05B with three biological repeats is presented in Table S1. After directly comparing the density and discrete distribution of the expression levels for different samples, we found that the sequencing quality was the same (Figure S1). Thus, the results indicated that the quality of the data obtained by sequencing was sufficient, and the data could be analyzed in the next step.

### 2.3. Functional Distribution of DEGs in 09-05A/B Lines of B. juncea

**Figure 3:**
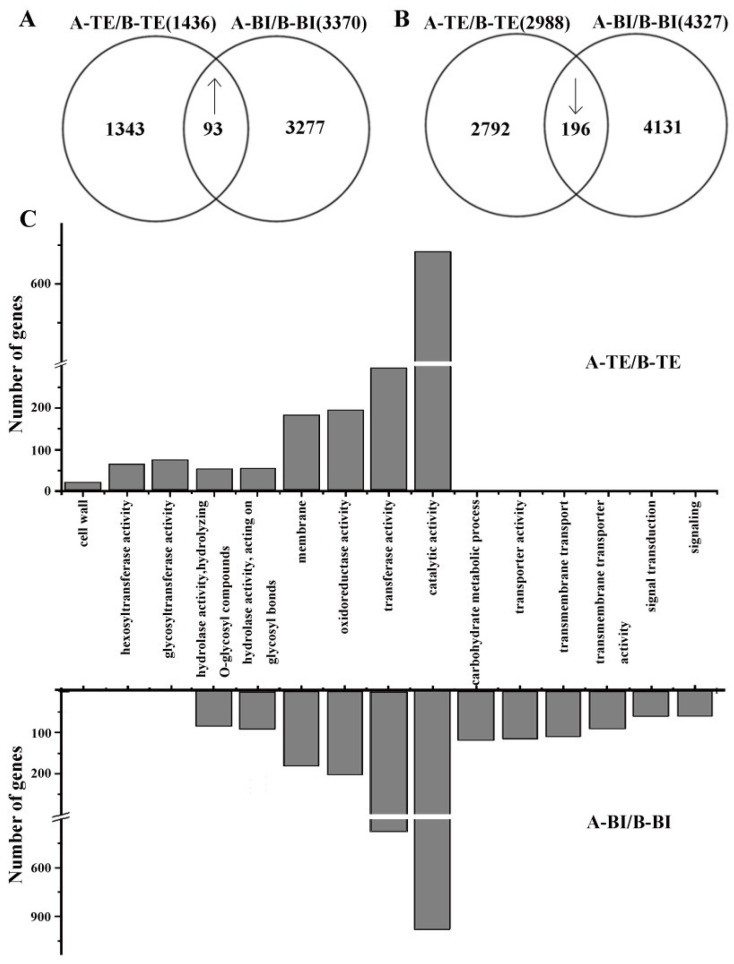
Functional distribution of identified genes in 09-05A and 09-05B flower buds at the TE and BI stages of *B. juncea*. RNAs were extracted, sequenced, and analyzed. Genes in flower buds in 09-05A were compared with those in 09-05B at the two stages, respectively. “↑” represents upregulation; “↓” represents downregulation. (**A**) Comparison of upregulated DEGs identified in 09-05A/09-05B. (**B**) Comparison of downregulated DEGs identified in 09-05A/09-05B. (**C**) Predicated and categorized functions of DEGs.

The expression level of each gene in 09-05A was compared with that in 09-05B separately at the TE and BI stages to explore the CMS molecular mechanism in 09-05A of *B. juncea*. Compared with 09-05B, a total of 11,832 DEGs at the TE and the BI stages were identified (Table S2), of which 289 DEGs (93 upregulated and 196 downregulated) were shared between the two groups (Figure 3A,B). A total of 1343 and 3277 upregulated genes were differentially expressed at the TE and BI stages, respectively (Figure 3A). A total of 2792 and 4131 downregulated genes were, respectively, identified at the TE and BI stages (Figure 3B). Notably, the number of DEGs showed an increasing trend at the BI stage compared with the TE stage, and more downregulated genes than upregulated genes were found at the two stages.

Based on functional analysis, DEGs related to hydrolase activity, membrane, oxidoreductase activity, transferase activity, and catalytic activity were differently enriched in 09-05A compared with 09-05B both at the TE and BI stages (Table S3). In addition, the number of genes related to those pathways in 09-05A was increased more at the BI stage than in 09-05B (Figure 3C). Furthermore, genes associated with cell wall, hexosyltransferase activity, and glycosyltransferase activity were specifically enriched in 09-05A compared with 09-05B at the TE stage. At the BI stage, DEGs were specifically annotated to the categories including hydrolase activity, carbohydrate metabolic process, transporter activity, transmembrane transport, transmembrane transporter activity, signal transduction, and signaling pathway in 09-05A compared with 09-05B. These results indicated that multiple complex metabolic pathways, enzyme activity, and transporter activity were involved in pollen development in 09-05A/B lines of *B. juncea*, and a remarkable difference was observed from the TE stage to the BI period of 09-05A/B lines, which was consistent with our cytological observations.

### 2.4. Functional Distribution of DAPs in 09-05A/B Lines of B. juncea

**Figure 4:**
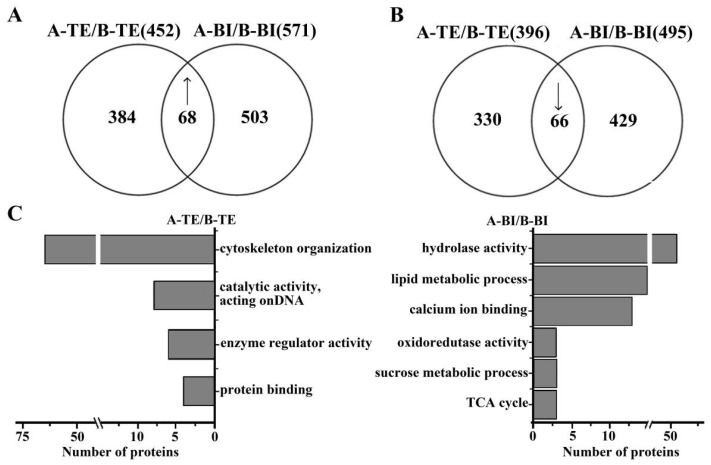
Functional distribution of DAPs in male sterile line 09-05A and its maintainer line 09-05B of *B. juncea*. Flower buds of *B. juncea* were collected at the TE and BI stages. Proteins were extracted, reduced, alkylated, digested, and analyzed by nanoLC-MS/MS. Proteins in flower buds of 09-05A were compared with those in 09-05B at the TE and BI stages, respectively. “↑” represents upregulation; “↓” represents downregulation. (**A**) Comparison of up-accumulated DAPs identified in 09-05A/09-05B. (**B**) Comparison of down-accumulated DAPs identified in 09-05A/09-05B. (**C**) Predicated and categorized functions of DAPs.

Label-free proteomic analysis was performed in 09-05A compared with 09-05B at the TE and BI stages to complement the transcriptomic study. The relative abundance of proteins from 09-05A was compared with that from 09-05B at two stages. A total of 955 upregulated and 825 downregulated proteins were identified in 09-05A compared with 09-05B at the TE and BI stages (Table S4). In addition, a total of 134 DAPs (68 upregulated and 66 downregulated) were shared among the two groups (Figure 4A,B). Among DAPs with upregulated expression, a total of 384 DAPs at the TE stage and 503 DAPs at the BI stage were identified in flower buds in 09-05A compared with 09-05B (Figure 4A). Among proteins with reduced expression, 330 and 429 DAPs were uniquely identified at the TE and BI stages, respectively (Figure 4B). Consistent with the RNA-seq data, the number of DAPs also showed an increasing trend at the BI stage.

Based on functional analysis, the DAPs in the 09-05A CMS line, which were related to protein binding, cytoskeleton organization, enzyme regulator activity, and catalytic activity at the TE stage compared with those in 09-05B (Figure 4C; Table S5). Furthermore, DAPs in 09-05A, which were related to hydrolase activity, sucrose metabolic process, oxidoreductase activity, calcium ion binding, lipid metabolic process, and TCA cycle, were particularly enriched at the BI stage in 09-05A compared with those in 09-05B (Figure 4C; Table S5).

### 2.5. MS Related DEGs and DAPs in Carbohydrate Metabolism and Energy Metabolism of 09-05A/B Lines of B. juncea

**Figure 5:**
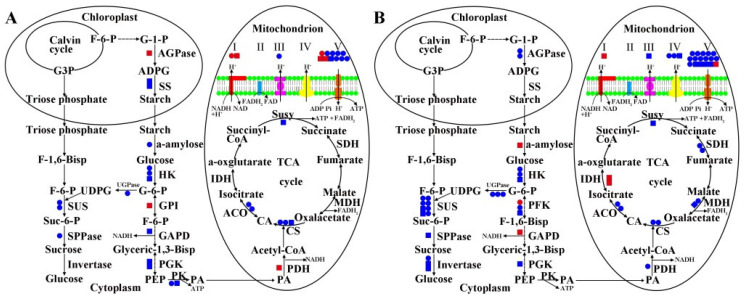
Integrated pathway of starch and sucrose metabolic process, glycolysis, TCA cycle, and respiratory chain pathway based on DEGs and DAPs identified in flower buds of *B. juncea*. (**A**) DEGs and DAPs involved in the integrated pathway in 09-05A/B lines at the TE stage. (**B**) DEGs and DAPs involved in the integrated pathway in 09-05A/B lines at the BI stage. DEGs and DAPs involved in these pathways in 09-05A were compared with those in 09-05B at the TE and BI stages, respectively. Red circle, blue circle, red square, and blue square indicate upregulated gene, downregulated gene, increased protein, and decreased protein, respectively. Abbreviations are as follows: ACO—aconitase; ADPG—adenosine diphosphate glucose; AGPase—ADP-glucose pyrophosphorylase; CA—citric acid; CS—citrate synthase; F-1-6-Bisp—Fructose-1-6-bisphosphate;—; F-6-P—fructose-6-phosphate; GAPD—glyceraldehyde-3-phosphate dehydrogenase; G-1-P—glucose-1-phosphate; G3P—glyceraldehyde 3-phosphate; G-6-P—glucose-6-phosphate; GPI—glucose-phosphate isomerase; HK—hexokinase; IDH—isocitrate dehydrogenase; MDH—malate dehydrogenase; PA—pyruvic acid; PDH—pyruvate dehydrogenase; PEP—phosphoenolpyruvate; PGK—phosphofructokinase; PK—pyruvate kinase; SDH—succinate dehydrogenase; SPPase—sucrose phosphate phosphatase; SS—starch synthase; Suc-6-P—sucrose-6-phosphate; SUS—sucrose synthase; Susy—succinyl-CoA synthases; UDPG—uridine diphosphate glucose; UGPase—UDP-glucosepyro phosphosphprylase.

Carbohydrates provide a material basis for anther and pollen development, and they are also an important component of the cell wall. In determining the exchange in carbohydrate metabolism of 09-05A, DEGs and DAPs related to carbohydrate metabolism were primarily mapped onto the starch, sucrose, and glycolysis pathways using the KEGG database (Table 1). The regulatory pattern of related DEGs and DAPs in the starch and sucrose metabolic pathways as well as the glycolysis pathway between 09-05A and 09-05B is shown in Figure 5. During starch synthesis, the protein quantity of starch synthase (SS) was decreased in 09-05A at the TE and BI stages compared with 09-05B. The protein quantity of ADP-glucose pyrophosphorylase (AGPase) was increased at the TE stage, but genes encoding AGPase were downregulated at the BI stage. During starch degradation in 09-05A compared with 09-05B, genes encoding α-amylose were downregulated, whereas the protein quantity of α-amylose was increased at the BI stage. During sucrose synthesis, the gene expression of sucrose synthase (SUS) and UDP-glucosepyro phosphosphprylase (UGPase) were downregulated at the TE and at BI stages in 09-05A compared with 09-05B. The gene expression and protein quantity of sucrose phosphate phosphatase (SPPase) were downregulated and decreased at the TE and BI stages, respectively. During sucrose degradation, the gene expression and protein quantity of invertase were downregulated and decreased in 09-05A at the BI stage.

In the glycolysis pathway, the key DEGs and DAPs were identified at the TE and BI stages in 09-05A compared with 09-05B. The gene expression and protein quantity of hexokinase (HK) were differentially downregulated and decreased at the TE and BI stages, respectively (Figure 5). The gene expression and protein quantity of 6-phosphofructokinase (PFK) were downregulated and decreased at the TE and BI stages, respectively. However, a gene-encoding PFK was upregulated at the BI stage. The gene expression and protein quantity of pyruvate kinase (PK) were downregulated and decreased at the TE stage. In addition, the expression of glyceraldehyde-3-phosphate dehydrogenase (GAPD) was downregulated at the TE stage but upregulated at the BI stage. Notably, the expression of pyruvate dehydrogenase (PDH) was increased at the TE stage, but the gene-encoding PDH was downregulated at the BI stage in the pyruvic acid (PA) biosynthesis pathway.

In investigating the exchange in energy metabolism in flower buds of 09-05A, the DEGs and DAPs related to energy metabolism were mapped onto the TCA cycle and oxidative phosphorylation pathways using the KEGG database (Table 2). Among TCA -cycle-related enzymes, citrate synthase (CS), aconitase (ACO), isocitrate dehydrogenase (IDH), succinyl-CoA synthase (SUC), succinate dehydrogenase (SDH), and malate dehydrogenase (MDH) differentially responded to MS in 09-05A compared with 09-05B at the TE and BI stages, respectively (Figure 5). Genes encoding ACO and MDH were downregulated at the TE and BI stages, respectively; the expression of MDH was decreased at the BI stage. The gene expression of CS was downregulated and decreased at the TE and BI stages, respectively; the protein quantity of CS was decreased at the TE stage. The protein quantity of SUC was decreased at TE and BI stages. Compared with 09-05B, genes encoding SDH and MDH were specifically downregulated in 09-05A at the BI stage. However, the protein quantity of IDH was increased at the BI stage.

We found that DEGs and DAPs involved in the oxidative phosphorylation pathway, including respiratory chain complex I (NADH dehydrogenase), cytochrome c, complex III (cytochrome reductase), complex IV (cytochrome oxidase), and complex V (ATP synthase), were primarily downregulated in 09-05A (Table 2). In addition, the protein quality and gene expression of complex I were increased and upregulated in 09-05A compared with 09-05B at the two stages. During this process, ATP was produced because of the participation of V-type proton ATPase, ADP, and Pi; H^+^ entered the membrane under the action of ATPase along the proton channel. The DEGs and DAPs were primarily downregulated in cytochrome reductase, cytochrome oxidase, and ATPase in 09-05A compared with 09-05B at the TE and BI stages. Thus, the rate of ATP production and H^+^ transport was influenced by downregulated genes encoding ATPase. These results indicated that 09-05A may have a decreased energy-generation capacity at the TE and BI stages.

### 2.6. Metabolic Products and Enzyme Activity Analyses in 09-05A/B Lines of B. juncea

**Figure 6:**
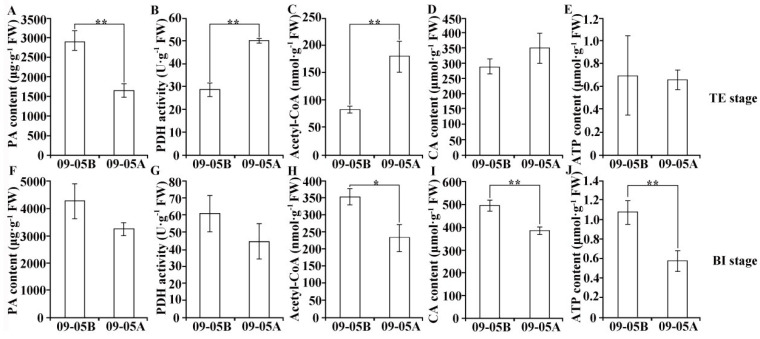
Determination of metabolic products and enzyme activity in 09-05A and 09-05B at the TE and BI stages of *B. juncea*. (**A**,**F**) Determination of PA content. (**B**,**G**) Determination of PDH activity. (**C**,**H**) Determination of acetyl-CoA content. (**D**,**I**) Determination of CA content. (**E**,**J**) Determination of ATP content. All data are shown as the mean ± SD from three independent biological replicates. CA—citric acid; FW—fresh weight; PA—pyruvic acid; PDH—pyruvate dehydrogenase. Significance at *p* < 0.01 level was marked with “**”; at *p* < 0.05 level it was marked with “*”; while at *p* > 0.05 level it was no marker which meant no significant difference between those samples.

The contents of PA, acetyl-CoA, CA, and ATP and the activity of PDH were measured in the buds of 09-05A and 09-05B at the TE and BI stages, respectively, to reveal the carbohydrate and energy metabolic pathways of *B. juncea* involved in MS (Figure 6). Compared with 09-05B, the results showed that the content of PA in the buds of 09-05A was differentially decreased by 43% and 24% at the TE and BI stages, respectively (Figure 6A,F). The activity of PDH in the buds of 09-05A significantly increased 1.7-fold compared with those of 09-05B at the TE stage but decreased by 25% at the BI stage (Figure 6B,G). The contents of acetyl-CoA and CA increased 2-fold and 1.2-fold at the TE stage in 09-05A compared with 09-05B but decreased by 34% and 22% at the BI stage, respectively (Figure 6C,D,H,I). The content of ATP slightly decreased with no evident difference at the TE stage but decreased by 46% at the BI stage in 09-05A compared with 09-05B (Figure 6E,J). These results indicated that the substance and energy metabolism in the buds of 09-05A were significantly affected particularly at the BI stage, which was consistent with transcriptomic and proteomic analyses (Figure 6).

## 3. Discussion

In flowering plants, the development of stamens and pollen requires high energy [29,30]. Carbohydrate and energy metabolism are basic metabolic pathways, which primarily provide energy and carbon sources [31]. In this study, the conjoint transcriptomic and proteomic analyses showed that most of the pathways included DEGs and DAPs related to carbohydrate and energy metabolism, such as starch and sucrose metabolism, glycolysis pathway, TCA cycle, and oxidative phosphorylation pathway (Table 1 and Table 2). In plants, sugar metabolism, including sugar biosynthesis, degradation, transport, and its regulation, plays an essential role in male reproduction [32]. Inhibiting enzymes in starch and sucrose metabolism decreases the amount of glucose entering glycolysis pathway. Consequently, this change will affect the TCA cycle by reducing PA content as a respiratory substrate. Damage of the TCA cycle will affect the mitochondrial respiratory chain indirectly and previous research has shown that defects in sugar metabolism and TCA cycle often result in MS [33]. Furthermore, when the respiratory chain is inhibited, excess electrons interact directly with oxygen molecules to produce ROS, which may trigger PCD to cause MS [34,35]. Therefore, our analysis provided important information for identifying genes/proteins related to MS and further exploring the molecular regulation mechanism of MS in 09-05A of *B. juncea*.

### 3.1. Microspores Are Defective with Degraded Cellular Components and Altered Pollen Wall in 09-05A

After the morphogenesis of the anther, the microspores are enclosed by the tapetum, which could secrete sporopollenin and provide essential nutrients for microspore development by secreting vesicles and self-degradation [36,37]. Studies have shown that the abnormal PCD of tapetum will affect pollen exine pattern formation and microspore development, thereby leading to pollen abortion and MS [38,39]. Che et al. [40] suggested that over-vacuolization and premature death could cause defective functions of the tapetum, which affected later anther development in the pepper sterile line. In addition, organelles, particularly the chloroplast and mitochondrion, play an essential role in microspore development. In chloroplasts, starch accumulation is necessary for microspore development, and it is a vital feature of fertile pollen [7]. Previous research has demonstrated that perturbed polysaccharide metabolism can lead to unusual starch storage and cause poor pollen wall formation [41,42]. The genes encoding glycosyltransferase enzyme families have also been reported to be associated with cell wall synthesis and degradation [43]. The mitochondrion is a crucial organelle for metabolic pathways such as respiratory electron transfer, ATP synthesis, and TCA cycle [44,45]. Considerable research has showed that the mitochondria might be involved in triggering death of male reproductive organs by affecting the level of ATP or ROS production [46]. In our study, the anthers of 09-05A compared with 09-05B became longer, and shriveled with no evident pollen grains on the surface (Figure 1C), and pollen grains were deformed because of the abnormal development at the BI stage (Figure 2G). Further TEM observation found that cellular components were evidently degraded with a defective pollen wall (Figure 2J) in 09-05A compared with 09-05B at the BI stage. Based on these results, we concluded that the abnormal degraded cellular components and morphologically altered pollen exine wall may cause the abnormalities of microspores and MS in 09-05A of *B. juncea*.

### 3.2. Damage to Starch–Sucrose Metabolism and Glycolysis Pathway May Inhibit the Production of Respiratory Substrate in 09-05A

During pollen development, sugars provide energy and nutrition for pollen maturation [43]. The absence of starch and sucrose in pollen grains was found to be associated with MS [47,48]. Recent analyses have also indicated that downregulated genes encoding sucrose synthesis, transport, and degradation, such as *SPP2*, *CsSUT1*, and invertase in a male sterile line, could trigger MS through the resultant perturbation in carbohydrate metabolism [43,49,50]. Among starch and sucrose metabolic pathways, four key enzymes, including AGPase, SS, UGPase, and invertase, have crucial roles in starch and sucrose metabolism. The research has shown that the lack of AGPase and SS in male sterile line might directly result in the reduction in starch, disturbing male reproduction and pollen sterility [51,52]. Based on previous reports, UGPase catalyzes the reversible production of glucose-1-phosphate (G-1-P) and UTP to UDP-glucose and pyrophosphate, and the inactivation of the UGPase1 gene may lead to MS in rice [53,54]. The significantly reduced activity of invertase can cause an inability to metabolize incoming sucrose to hexoses, which may lead to pollen-developmental lesion in the wheat K-CMS line [55]. In addition, the glycolysis pathway is used as a respiratory substrate through the conversion of glucose to PA [56]. HK catalyzes the first step in glycolysis, and deficiency of hexokinase *HXK5* impairs the utilization of starch in pollen grains and cause MS in rice [57]. PK plays a role in regulating cell metabolism by catalyzing the conversion of phosphoenolpyruvate (PEP) and ADP to ATP and PA as a respiratory substrate in glycolysis [58]. In a previous study, the downregulation of a PK-responsive gene might decrease carbohydrate accumulation in the flowers of broccoli CMS line [59]. In our study, a series of DEGs/DAPs, including the four aforementioned key enzymes that regulate the metabolism of starch and sucrose used for energy supply, was downregulated in 09-05A compared with 09-05B (Figure 5 and Table 1). In addition, HK, PFK, and PK, which were included in glycolysis as rate-limiting enzymes, catalyzing irreversible chemical reactions, were differentially downregulated in 09-05A (Table 1). The conjoint analysis also showed that the protein quantity of phosphofructokinase (PGK) was decreased in 09-05A (Figure 5). Furthermore, we measured the content of PA found in the buds of 09-05A were significantly lower than that of 09-05B at the TE and BI stages (Figure 6A). These findings indicated that the downregulation of key enzymes involved in the starch–sucrose metabolism and glycolysis pathway may greatly reduce the amount of PA as a respiratory substrate entering the TCA cycle, thereby affecting the mitochondrial respiratory chain indirectly and probably inducing MS in 09-05A of *B. juncea*.

### 3.3. Damage to the TCA Cycle and Respiratory Chain May Inhibit the Production of ATP in 09-05A

In previous studies, defects in the TCA cycle may cause MS [60]. Among these enzymes, PDH plays a role in regulating cell metabolism by catalyzing the conversion of PA to acetyl-CoA and NADH entering TCA [58]. A previous study has shown that inhibiting the activity of PDH in anthers could cause MS in sugar beet [61]. CS is the initial enzyme of the TCA cycle, which plays an essential role in energy supply for pollen development [62]. In pepper, transcriptional analysis revealed that the gene expression of CS in anthers of the CMS line was lower than that in the maintainer [63]. IDH catalyzes the oxidative decarboxylation of isocitrate to produce α-ketoglutarate and CO_2_, which is an irreversible and rate-limiting step in the TCA cycle. In addition, dysfunctional SDH could lead to abnormal gametophyte development, aborted pollen, and decreased seed quantity in *Arabidopsis* [64]. Mitochondrial ATP synthesis is driven by electron transport in the inner membrane and the demand for ATP is highly increased during pollen development in higher plants. NADH dehydrogenase is the first enzyme in the mitochondrial electron transfer chain, which is a major site of premature electron leakage to oxygen, and it plays a significant role in triggering apoptosis [65,66]. Previous studies have shown that the alteration of mitochondrial-encoded subunits of ATPase may inhibit ATP production and induce MS in plants [67]. In our present analysis, other downregulated enzymes apart from CS and SDH, including ACO, succinyl-CoA synthases (Susy), and MDH, were identified in the TCA cycle (Figure 5). Moreover, we found that the protein quantity of IDH was increased in 09-05A at the BI stage, which was consistent with the results of a previous study; that is, the overexpression of the maize IDH gene *Zm00001d008244* could disturb plant fertility [68]. Furthermore, the activity of PDH in the buds of 09-05A was decreased at the TE stage compared with those of 09-05B (Figure 6B). These studies indicate that all of these changes may have reduced the amount of coenzymes (NADH and FADH_2_) produced in the TCA cycle; thus, fewer coenzymes will enter the respiratory chain, and the formation of ATP will decrease. Most DEGs and DAPs regulating cytochrome reductase, cytochrome oxidase, and ATPase were downregulated in the respiratory chain in 09-05A compared with 09-05B (Table 2). Moreover, we found that the content of ATP was increasingly reduced at the BI stage in 09-05A (Figure 6J), and many studies have detected lower ATP production in some CMS flowers [69,70]. Therefore, the change in the abundance of these proteins might result in abnormal energy metabolism pathway, which leads to an insufficient supply of critical substrates, thereby reducing the efficiency of the energy supply during pollen development, and leading to MS in 09-05A of *B. juncea*.

## 4. Materials and Methods

### 4.1. Plant Materials and Growth Conditions

The CMS line 09-05A and its maintainer line 09-05B were cultivated in the same experimental plot in Ningbo Academy of Agricultural Sciences, Zhejiang, China. The plants were grown in the greenhouse (25 cm × 20 cm in plant spacing) under the normal environmental conditions with regular fertilizer and water management. This CMS line 09-05A was developed via multiple backcrosses between the winter stem mustard *oxa* CMS line and spring stem mustard maintainer line. After flowering, the sterile and fertile plants were identified, and the buds at the TE and BI stages were collected (three biological replicates) from the individual plants for transcriptomic and proteomic analyses. The collected plant materials were frozen in liquid nitrogen and stored at −80 °C for further analysis.

### 4.2. Morphological and Cytological Observation

Floral buds at the TE and BI stages separately from 09-05A and 09-05B were fixed with 2.5% glutaraldehyde in phosphate buffer (pH 7.0) overnight, and post-fixed with 1% OsO_4_ in phosphate buffer for 1 h. Then, the specimens were dehydrated through a graded series of ethanol and embedded in Spurr resin. Semi-thin sections (1 μm) were sliced under an ultramicrotome (LKB 11800, Stockholm, Sweden), stained with 0.5% toluidine blue, and photographed with a fluorescence microscope (DMLB, Leica, Germany). For TEM, ultrathin sections (70 nm) were obtained and stained with uranyl acetate, followed by alkaline lead citrate, and observed in a transmission electron microscope (H-7650, Hitachi, Japan).

### 4.3. RNA Extraction for Transcriptomic Analysis

Total RNA was isolated from 100 mg of buds from 09-05A and 09-05B at the TE and BI stages, respectively, using the RNAprep pure Plant Kit (Tiangen Biotech, Beijing, China) according to the supplier’s instruction. Total RNA was treated with RNase-free DNase I (Tiangen Biotech). Three independent biological replicates for each sample were included. DNA-free total RNA was used for Illumina Tru-seq library preparation according to the manufacturer’s instruction. RNAs from three biological replicates were sequenced separately at Beijing Novogene Bioinformatics Technology Co., Ltd. (Beijing, China), using Illumina Hiseq X-Ten.

### 4.4. Transcriptomic Sequencing, Data Processing, and Transcriptomic Analysis

Total RNA was submitted to Novogene in Beijing (Novogene. https://en.novogene.com/; accessed on 1 May 2022) for library construction and RNA sequencing. Messenger RNA was enriched by oligo(dT)-attached magnetic beads for cDNA synthesis. Then, size-selected and adaptor-ligated cDNA fragments were purified for library construction. The cleaved RNA fragments by fragmentation buffer were transcribed into first-strand cDNA using reverse transcriptase and random hexamer primers. Subsequently, second-strand cDNA synthesis was performed using DNA polymerase I and RNase H. After purification and end-repair, the fragments were ligated to sequence adaptors and amplified by polymerase chain reaction (PCR). PCR products were purified with AMPure XP system (Beckman Coulter, Beverly, CA, USA) Amplified cDNA libraries were evaluated by using an Agilent 2100 Bioanalyzer (Agilent, Palo Alto, CA, USA). Library preparations were sequenced on an Illumina HiSeq X-Ten platform and 150 bp paired-end reads were generated.

Raw reads were cleaned by removing reads containing adapter, ploy-N, and low-quality reads from raw data. The Q20 (the percentage of bases with a Phred value of >20), Q30 (the percentage of bases with a Phred value of >20), and GC (base G and C) content of clean data was calculated. All downstream analyses were based on clean, high-quality data. All clean reads were mapped onto *B. juncea* reference genomes (*Brassica juncea*. http://brassicadb.org/brad/datasets/pub/Genomes/Brassica_juncea/V1.5/; accessed on 1 May 2022). An index of the reference genome was built, and paired-end clean reads were aligned to the reference genome using Hisat2 v2.0.5. FragmentsPer Kilobase Per Million (FPKM) was used to show the expression value. The fold-change was calculated by 09-05A/09-05B. DESeq2R package was applied to identify the DEGs with a *p*-value of < 0.05 and fold-change of > 1.5 or < 0.667.

### 4.5. Total Protein Extraction and Peptide Preparation

In brief, samples were individually milled to powder in a mortar with liquid nitrogen and then mixed with lysis buffer (containing 50 mM Tris-HCl−pH 8, 8 M urea, and 0.2% SDS). Then, the homogenate was incubated through ultrasonication on ice for 5 min and centrifuged at 12,000× *g* for 15 min at 4 °C. Protein concentration was determined using a Bradford assay (Beyotime Biotechnology, Shanghai, China) after transferring the supernatant to a clean tube. The supernatant was reduced by adding 2 mM of DTT at 56 °C for 1 h. Afterward, sufficient iodoacetic acid was added to the sample, and the mixture was incubated in darkness for 1 h. Next, sufficient iodoacetamide was added to the sample, and the mixture was incubated for 1h at room temperature in the dark. A fourfold volume of precooled acetone was mixed with the samples and incubated at −20 °C for at least 2 h; the samples were then centrifuged to collect precipitation. The extracts were centrifuged at 12,000× *g* for 15 min at 4 °C. The pellets were collected, washed two times with cold acetone, and dissolved in a buffer containing 0.1 M triethylammonium bicarbonate (TEAB, pH 8.5) and 8 M urea. Protein concentration was determined using the Bradford assay (Beyotime Biotechnology) with bovine serum albumin as the standard. Supernatant from each sample containing 0.1 mg of protein was digested with Trypsin Gold (Promega, Madison, WI, USA) at 37 °C for 16 h. The peptide was dried by vacuum centrifugation after the removal of urea using a C18 desalting cartridge.

### 4.6. LC–MS/MS Analysis

LC–MS/MS analyses were performed using an Orbitrap Q Exactive HF-X mass spectrometer (Thermo Fisher Scientific, Bremen, Germany) combined with an EASY-nLC™ 1200 UHPLC system (Thermo Fisher Scientific, Waltham, MA, USA). The dried fractions were resuspended in 0.1% formic acid (FA) and then loaded onto an Acclaim PepMap 100 C18 Nano-Trap column (2 cm × 100 μm, 5 μm). Peptides were separated on a Reprosil-Pur 120 C18-AQ analytical column (15 cm × 150 μm, 1.9 μm) using a 60 min linear gradient from 5% to 100% eluent B (0.1% FA in 80% acetonitrile in eluent A (0.1% FA in H_2_O) at a flow rate of 600 nL/min. The solvent gradient was as follows: 5–10% B, 2 min; 10–30% B, 49 min; 30–50% B, 5 min; 50–90% B, 1 min; and 90–100% B, 5 min.

The separated peptides were analyzed by using a Q Exactive HF-X mass spectrometer (Thermo Fisher Scientific, Bremen, Germany) equipped with a Nanospray Flex™ iron sucrose (ESI), spray voltage of 2.3 kV, and ion transport capillary temperature of 320 °C. Full MS scans from 350 to 1500 m/z were acquired at a resolution of 60,000 (at 200 m/z), automatic gain control (AGC) target value of 3 × 10^6^, and a maximum ion injection time of 20 ms. Based on the full MS scan, 40 of the abundant precursor ions were selected for high-energy collisional dissociation fragment analysis at a resolution of 15,000 (at 200 m/z), an AGC target value of 1 × 10^5^, a maximum ion injection time of 45 ms, a normalized collision energy of 27%, an intensity threshold of 2.2 × 10^4^, and a dynamic exclusion parameter of 20 s.

### 4.7. Protein Identification from Mass Spectrometry Data

Proteins were identified using Proteome Discoverer 2.2 (PD 2.2, ThermoFisher Scientific) with *B. juncea* database (*Brassica juncea*. http://brassicadb.org/brad/datasets/pub/Genomes/Brassica_juncea/V1.5/; accessed on 1 May 2022). The search parameters were set as follows: a mass tolerance for precursor ion scans and product ion scans were 10 ppm and 0.02 Da, respectively. Carbamidomethyl was used as fixed modifications in PD 2.2. Variable modifications in PD 2.2 included lysine, N-terminus acetylation, and methionine oxidation. With regard to identification, at the peptide and protein levels, the false discovery rate was less than 1.0%, and proteins were identified with at least one unique peptide. DEPs between 09-05A and 09-05B samples were identified in accordance with the criteria of *p*-value of less than 0.05 and fold-change of >1.5 (significantly upregulated) or fold-change of <0.667 (significantly downregulated).

### 4.8. Functional Annotation

A summary of GO annotation categories, including molecular function, biological process and cellular component, was generated using TBtools made in-house (TBtools. https://github.com/CJ-Chen/TBtools/releases; accessed on 1 May 2022). Pathway mapping of identified proteins and genes was performed using a genomes (KEGG) database (KEGG. http://www.genome.jp/kegg/; accessed on 1 May 2022).

### 4.9. Metabolic Products and Enzyme Activity Analyses

The content of PA, acetyl-CoA, and ATP was measured using a commercially available PA Content Assay Kit (Geruisi, Suzhou, China), acetyl-CoA Content Assay Kit (Geruisi, Suzhou, China), and ATP Content Assay Kit (Geruisi, Suzhou, China). In brief, flower buds were ground into a homogenate and then centrifuged for 10 min at 12,000 rpm and 4 °C. The absorbance of the collected supernatant of PA, acetyl-CoA, and ATP was measured at 520, 340, and 700 nm using UV spectrophotometry, respectively. Total PA, acetyl-CoA, and ATP levels were expressed as μg∙g^−1^, nmol∙g^−1^, and μmol∙g^−1^ fresh weight (FW), and three biological replicates were performed.

The content of CA and PDH activity were assayed using a CA Assay Kit (Solarbio, Beijing, China) and a PDH Assay Kit (Solarbio, Beijing, China), respectively. In brief, flower buds were ground into a homogenate and then centrifuged for 10 min at 11,000× *g* and 4 °C. The absorbance of the collected supernatant of CA and PDH was measured at 545 and 605 nm using UV spectrophotometry, respectively. Total CA and PDH levels were expressed separately as μmol∙g^−1^ and U∙g^−1^ FW. The experiments were repeated three times.

### 4.10. Statistical Analysis

SPSS statistical software (version 22.0; IBM, Armonk, NY, USA) was used for statistical evaluation. Statistical significance was evaluated by Student’s *t*-test or one-way ANOVA when only two groups were compared. A *p*-value of less than 0.05 was considered statistically significant.

## 5. Conclusions

In exploring the molecular mechanism of MS of *B. juncea*, the buds at the TE and BI stages of 09-05A and its maintainer line 09-05B were collected for transcriptomic and proteomic analyses. A total of 11,832 transcripts and 1780 protein species were identified to have a significantly changed pattern at the transcriptomic and proteomic levels, respectively. Functional annotation analyses indicated that these DEGs/DEPs were involved in flower development and pollen development, and they may be related to the reduction in fertility in *B. juncea*. Several key biological processes such as sucrose metabolism, TCA cycle, and oxidoreductase activity were found to be closely related to MS in 09-05A. Among these pathways, the downregulated expression of key genes/proteins, leading to an insufficient supply of critical substrates and ATP, might result in the damage of carbohydrate and energy metabolism and eventually lead to MS in 09-05A. Therefore, the conjoint analysis of the transcriptome and proteome will improve our understanding of genes and pathways associated with MS in *B. juncea* and provide insights into the molecular regulatory mechanism of MS in plants.

## Figures and Tables

**Table 1:** List of DEGs and DAPs associated with carbohydrate metabolism in 09-05A/B lines of *B. juncea*.

TE Stage	Gene ID	KO ID	KO Description	Annotation	Regulation
DEG	BjuA029507	K01176	alpha-amylase	-	DOWN
DEG	BjuB037515	K00695	sucrose synthase	AT5G49190	DOWN
DEG	BjuB030962	K00695	sucrose synthase	-	DOWN
DEG	BjuO008945	K00695	sucrose synthase	-	DOWN
DEG	BjuA046136	K00963	UTP--glucose-1-phosphate uridylyltransferase	-	DOWN
DEG	BjuB005071	K07024	sucrose-6-phosphatase	--	DOWN
DEG	BjuB047043	K00844	hexokinase	AT1G50460	DOWN
DEG	BjuA021412	K00844	hexokinase	-	DOWN
DEG	novel.400	K00850	6-phosphofructokinase 1	AT4G26270	DOWN
DEG	BjuB040267	K00873	pyruvate kinase	AT3G49160	DOWN
DAP	BjuA041438	K00975	Glucose-1-phosphate adenylyltransferase large subunit 1	AT5G19220	UP
DAP	BjuA023577	K00703	Starch synthase, chloroplastic/amyloplastic	AT5G24300	DOWN
DAP	BjuA037309	K00844	Phosphotransferase	AT1G47840	DOWN
DAP	BjuB028137	K01810	Glucose-6-phosphate isomerase	AT5G42740	UP
DAP	BjuA006306	K00134	Glyceraldehyde-3-phosphate dehydrogenase	AT1G13440	DOWN
DAP	BjuA032999	K00927	Phosphoglycerate kinase	AT1G79550	DOWN
DAP	BjuB022100	K00927	Phosphoglycerate kinase	AT1G79550	DOWN
DAP	BjuA006685	K00873	Pyruvate kinase	AT5G63680	DOWN
**BI Stage**	**Gene ID**	**KO ID**	**KO Description**	**Annotation**	**Regulation**
DEG	BjuB038490	K00975	glucose-1-phosphate adenylyltransferase	AT4G39210	DOWN
DEG	BjuB030220	K00695	sucrose synthase	AT1G73370	DOWN
DEG	BjuO006586	K00695	sucrose synthase	AT4G02280	DOWN
DEG	BjuB015313	K00695	sucrose synthase	AT5G20830	DOWN
DEG	BjuB037515	K00695	sucrose synthase	AT5G49190	DOWN
DEG	BjuB030962	K00695	sucrose synthase	-	DOWN
DEG	BjuO008945	K00695	sucrose synthase	-	DOWN
DEG	BjuA041856	K00963	UTP--glucose-1-phosphate uridylyltransferase	AT5G17310	DOWN
DEG	BjuA046136	K00963	UTP--glucose-1-phosphate uridylyltransferase	-	DOWN
DEG	BjuO002531	K01193	beta-fructofuranosidase	AT2G36190	DOWN
DEG	BjuB047043	K00844	hexokinase	AT1G50460	DOWN
DEG	BjuA021412	K00844	hexokinase	-	DOWN
DEG	novel.6397	K00850	6-phosphofructokinase 1	AT5G56630	UP
DEG	BjuB016068	K00850	6-phosphofructokinase 1	-	DOWN
DAP	BjuB038082	K00703	Starch synthase, chloroplastic/amyloplastic	AT5G24300	DOWN
DAP	BjuB030962	K00695	Sucrose synthase	AT1G73370	DOWN
DAP	BjuO008945	K00695	Sucrose synthase	AT5G20830	DOWN
DAP	BjuO007590	K07024	SPP1	AT1G51420	DOWN
DAP	BjuO002531	K01193	CwINV4	AT2G36190	DOWN
DAP	BjuB026743	K01193	Beta-fructofuranosidase	AT2G36190	DOWN
DAP	BjuB042433	K00134	Gp_dh_N domain-containing protein	AT3G04120	UP
DAP	BjuB022100	K00927	Phosphoglycerate kinase	AT1G79550	DOWN
DAP	BjuB048068	K19893	X8 domain-containing protein	AT5G58090	UP

Gene ID, according to *B. juncea* database. Annotation, according to *Arabidopsis thaliana* database. “-”—no homologous gene in *A. thaliana* database. UP—A/B fold-change > 1.5; DOWN—A/B fold-change < 0.667.

**Table 2:** List of DEGs and DAPs associated with energy metabolism in 09-05A/B lines of *B. juncea*.

TE Stage	Gene ID	KO ID	KO Description	Annotation	Regulation
DEG	BjuA041635	K03940	NADH dehydrogenase (ubiquinone)	AT5G11770	UP
DEG	BjuA017841	K01647	citrate synthase	-	DOWN
DEG	novel.10587	K01647	citrate synthase	AT2G42790	DOWN
DEG	BjuA012893	K01681	aconitate hydratase	-	DOWN
DEG	BjuB043684	K01681	aconitate hydratase	AT4G26970	DOWN
DEG	BjuA015174	K00417	ubiquinol-cytochrome c reductase subunit 7	AT5G25450	DOWN
DEG	novel.1027	K01535	H^+^—transporting ATPase	-	DOWN
DEG	BjuA047355	K02133	F-type H^+^—transporting ATPase subunit beta	-	DOWN
DEG	BjuB036256	K02133	F-type H^+^—transporting ATPase subunit beta	AT5G08690	DOWN
DEG	BjuA033276	K02150	V-type H^+^—transporting ATPase subunit E	-	DOWN
DEG	BjuB029476	K02150	V-type H^+^—transporting ATPase subunit E	AT3G08560	DOWN
DEG	BjuO006984	K02154	V-type H^+^—transporting ATPase subunit a	-	DOWN
DEG	novel.8077	K02154	V-type H^+^—transporting ATPase subunit a	-	DOWN
DAP	BjuB029356	K00627	Acetyltransferase component of pyruvate	AT1G54220	UP
DAP	BjuB040953	K01899	dehydrogenase complex	AT5G08300	DOWN
DAP	BjuO005963	K01214	Succinate—CoA ligase	AT2G39930	DOWN
DAP	BjuA013768	K02267	ISA1	AT5G57815	UP
DAP	BjuB028190	K03953	Cytochrome c oxidase subunit	AT2G20360	UP
DAP	BjuB004697	K02154	V-type proton ATPase subunit a	AT4G39080	DOWN
DAP	BjuA015211	K02138	ATP synthase subunit d, mitochondrial	AT3G52300	UP
DAP	BjuO008600	K02154	V-type proton ATPase subunit a	AT4G39080	UP
**BI Stage**	**Gene ID**	**KO ID**	**KO Description**	**Annotation**	**Regulation**
DEG	BjuA038905	K00627	pyruvate dehydrogenase E2 component	-	DOWN
DEG	BjuA017841	K01647	citrate synthase	-	DOWN
DEG	novel.10587	K01647	citrate synthase	AT2G42790	DOWN
DEG	BjuA015968	K00026	malate dehydrogenase	AT2G22780	DOWN
DEG	BjuA023314	K00234	succinate dehydrogenase	AT2G18450	DOWN
DEG	BjuA026416	K00235	succinate dehydrogenase	AT5G40650	DOWN
DEG	BjuB043684	K01681	aconitate hydratase	AT4G26970	DOWN
DEG	BjuA012893	K01681	aconitate hydratase	-	DOWN
DEG	novel.1027	K01535	H^+^—transporting ATPase	-	DOWN
DEG	BjuA047355	K02133	F-type H^+^—transporting ATPase subunit beta	-	DOWN
DEG	BjuB036256	K02133	F-type H^+^—transporting ATPase subunit beta	AT5G08690	DOWN
DEG	BjuA033276	K02150	V-type H^+^—transporting ATPase subunit E	-	DOWN
DEG	BjuB029476	K02150	V-type H^+^—transporting ATPase subunit E	AT3G08560	DOWN
DEG	BjuO006984	K02154	V-type H^+^—transporting ATPase subunit a	-	DOWN
DEG	novel.8077	K02154	V-type H^+^—transporting ATPase subunit a	-	DOWN
DEG	novel.10278	K02146	V-type H^+^—transporting ATPase subunit d	-	DOWN
DEG	novel.4259	K02147	V-type H^+^—transporting ATPase subunit B	-	DOWN
DEG	BjuA013499	K02152	V-type H^+^—transporting ATPase subunit G	-	DOWN
DEG	BjuB014179	K02152	V-type H^+^—transporting ATPase subunit G	-	DOWN
DEG	BjuB048951	K02152	V-type H^+^—transporting ATPase subunit G	-	DOWN
DEG	novel.397	K02152	V-type H^+^—transporting ATPase subunit G	-	DOWN
DAP	BjuA004114	K02152	V-type H^+^—transporting ATPase subunit G	-	DOWN
DAP	BjuA029659	K00030	isocitrate dehydrogenase (NAD^+^)	AT4G35650	DOWN
DAP	BjuB024174	K00026	Malate dehydrogenase	AT2G22780	DOWN
DAP	BjuA003018	K00030	IDH1	AT4G35260	UP
DAP	BjuA003018	K00030	IDH1	AT4G35260	UP
DAP	BjuB019661	K00026	Malate dehydrogenase, chloroplastic	AT3G47520	UP
DAP	BjuB040953	K01899	Succinate—CoA ligase	AT5G08300	DOWN
DAP	BjuB027452	K03963	Uncharacterized protein	AT2G02050	UP
DAP	BjuA012602	K03966	Uncharacterized protein	AT3G18410	UP
DAP	BjuA004528	K03966	Uncharacterized protein	AT3G18410	UP
DAP	BjuB003649	K02266	Uncharacterized protein	AT4G37830	DOWN
DAP	BjuA001747	K02152	V-type proton ATPase subunit G	AT4G23710	UP
DAP	BjuB004697	K02154	V-type proton ATPase subunit a	AT4G39080	DOWN
DAP	BjuO008600	K02144	V-type proton ATPase subunit a	AT4G39080	DOWN
DAP	BjuA034730	K02150	VHA-E2	AT3G08560	DOWN
DAP	BjuB029476	K02150	VHA-E2	AT3G08560	DOWN

Gene ID, according to *B. juncea* database. Annotation, according to *Arabidopsis thaliana* database. “-”—no homologous gene in *A. thaliana* database. UP—A/B fold-change > 1.5; DOWN—A/B fold-change < 0.667.

## Supplementary Materials

The following supporting information can be downloaded at: https://www.mdpi.com/article/10.3390/ijms23116248/s1.
