# Supplementary material for: Integrative Analysis of Transcriptomic and Proteomic Changes Related to Cytoplasmic Male Sterility in Spring Stem Mustard (Brassica juncea var. tumida Tsen et Lee)
Source: Int J Mol Sci. 2022 Jun 2;23(11):6248. doi: 10.3390/ijms23116248 (PMC9180981; doi:10.3390/ijms23116248)
Supplement: Supplementary file 1 [file ijms-23-06248-s001.zip › Figure S1.pdf]

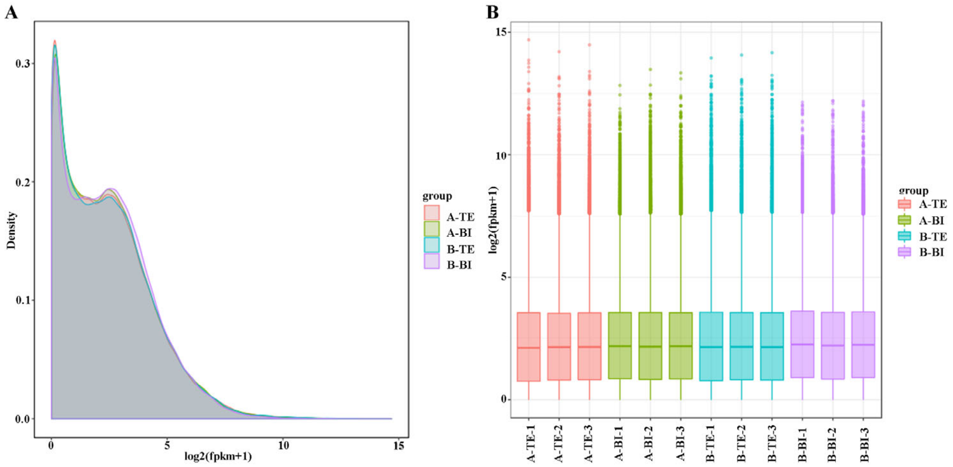

Figure S1. FPKM density distribution curve and box plot of each sample. (A) The curves of different colors in the figure represent different samples, the abscissa of the points on the curve represents the logarithm of the corresponding sample FPKM, and the ordinate of the points represents the probability density. (B) The abscissa is the sample name, the ordinate is $\log_2$ (FPKM+1), and the box graph pairs of each region have five statistics (the maximum, upper quartile, median, lower quartile, and minimum from top to bottom).
