# Supplementary material for: Integrative Analysis of Transcriptomic and Proteomic Changes Related to Cytoplasmic Male Sterility in Spring Stem Mustard (Brassica juncea var. tumida Tsen et Lee)
Source: Int J Mol Sci. 2022 Jun 2;23(11):6248. doi: 10.3390/ijms23116248 (PMC9180981; doi:10.3390/ijms23116248)
Supplement: Supplementary file 1 [file ijms-23-06248-s001.zip › Table S1.pdf]

Table S1. Statistics of RNA-seq analysis cover two stages of pollen development: 09-05B pollen of the TE stage (B-TE), 09-05B pollen of the BI stage (B-BI), 09-05A pollen of the TE stage (A-TE) and 09-05A pollen of the TE stage (A-BI) libraries including three biological replicates and assembly for *B. juncea*.

**Sample**	**Total reads**	**Q30**	**GC content**	**Total mapped**	**Multiple mapped**	**Unique mapped**
B-TE_1	46268762	93.75	47.24	44166788(95.46%)	5024175(10.86%)	39142613(84.6%)
B-TE_2	45878248	93.65	47.21	43730551(95.32%)	4890810(10.66%)	38839741(84.66%)
B-TE_3	45836602	92.7	46.87	43568969(95.05%)	4907489(10.71%)	38661480(84.35%)
B-BI_1	45593102	93.66	46.41	41912135(91.93%)	4959005(10.88%)	36953130(81.05%)
B-BI_2	46158438	95.7	46.57	43216083(93.63%)	5138978(11.13%)	38077105(82.49%)
B-BI_3	46293678	92.87	46.07	42748439(92.34%)	5038490(10.88%)	37709949(81.46%)
A-TE_1	43534400	95.71	45.94	41584300(95.52%)	4434931(10.19%)	37149369(85.33%)
A-TE_2	49958152	93.56	47.07	47277708(94.63%)	5305367(10.62%)	41972341(84.01%)
A-TE_3	45861062	92.5	46.17	43301437(94.42%)	4707316(10.26%)	38594121(84.15%)
A-BI_1	46178982	94.56	47.09	43953236(95.18%)	4924837(10.66%)	39028399(84.52%)
A-BI_2	45915878	93.41	47.41	43653645(95.07%)	5036611(10.97%)	38617034(84.1%)
A-BI_3	44816650	92.68	46.63	42491659(94.81%)	4738146(10.57%)	37753513(84.24%)
Total	552293954	93.73	46.72	521604950(94.45%)	59106155(10.70%)	462498795(83.75%)
